# Metagenomic next-generation sequencing could play a pivotal role in validating the diagnosis of invasive mold disease of the central nervous system

**DOI:** 10.3389/fcimb.2024.1393242

**Published:** 2024-06-07

**Authors:** Erhu Wei, Jiechao Niu, Mengjiao Zhang, Yu Zhang, Kunli Yan, Xiao Fang, Wei Ma, Lei Xie, Peisheng Jia, Huaili Wang

**Affiliations:** Department of Pediatrics, The First Affiliated Hospital of Zhengzhou University, Zhengzhou, China

**Keywords:** invasive mold disease, *Aspergillus fumigatus*, *Rhizomucor miehei*, *Rhizomucor pusillus*, metagenomic next-generation sequencing

## Abstract

**Background:**

Invasive mold diseases of the central nervous (CNS IMD) system are exceedingly rare disorders, characterized by nonspecific clinical symptoms. This results in significant diagnostic challenges, often leading to delayed diagnosis and the risk of misdiagnosis for patients. Metagenomic Next-Generation Sequencing (mNGS) holds significant importance for the diagnosis of infectious diseases, especially in the rapid and accurate identification of rare and difficult-to-culture pathogens. Therefore, this study aims to explore the clinical characteristics of invasive mold disease of CNS IMD in children and assess the effectiveness of mNGS technology in diagnosing CNS IMD.

**Methods:**

Three pediatric patients diagnosed with Invasive mold disease brain abscess and treated in the Pediatric Intensive Care Unit (PICU) of the First Affiliated Hospital of Zhengzhou University from January 2020 to December 2023 were selected for this study.

**Results:**

Case 1, a 6-year-old girl, was admitted to the hospital with “acute liver failure.” During her hospital stay, she developed fever, irritability, and seizures. CSF mNGS testing resulted in a negative outcome. Multiple brain abscesses were drained, and *Aspergillus fumigatus* was detected in pus culture and mNGS. The condition gradually improved after treatment with voriconazole combined with caspofungin. Case 2, a 3-year-old girl, was admitted with “acute B-lymphoblastic leukemia.” During induction chemotherapy, she developed fever and seizures. *Aspergillus fumigatus* was detected in the intracranial abscess fluid by mNGS, and the condition gradually improved after treatment with voriconazole combined with caspofungin, followed by “right-sided brain abscess drainage surgery.” Case 3, a 7-year-old girl, showed lethargy, fever, and right-sided limb weakness during the pending chemotherapy period for acute B-lymphoblastic leukemia. *Rhizomucor miehei* and *Rhizomucor pusillus* was detected in the cerebrospinal fluid by mNGS. The condition gradually improved after treatment with amphotericin B combined with posaconazole. After a six-month follow-up post-discharge, the three patients improved without residual neurological sequelae, and the primary diseases were in complete remission.

**Conclusion:**

The clinical manifestations of CNS IMD lack specificity. Early mNGS can assist in identifying the pathogen, providing a basis for definitive diagnosis. Combined surgical treatment when necessary can help improve prognosis.

## 1 Introduction

Invasive mold disease (IMD) presents a significant challenge in the intensive care unit, with a high incidence and mortality rate among immunocompromised children (Bassetti and Bouza, 2017). Invasive *aspergillosis* and *mucormycosis* are the two most common types of IMD, with mortality rates exceeding 30% even with appropriate treatment. The most common site of IMD infection is the lungs, while central nervous system invasive mold disease (CNS IMD) is less common, accounting for only 1% of all cases (Egger et al., 2023). Due to the nonspecific clinical features of CNS IMD and the lack of efficient diagnostic methods, diagnoses are often delayed.

Metagenomic next-generation sequencing (mNGS) can detect all potential microbes in pediatric samples, offering advantages over traditional culture methods, such as shorter detection cycles, wider detection ranges, and higher positivity rates. It is particularly suitable for cases where traditional culture methods cannot identify the pathogen or when rapid identification of the pathogen is necessary (Forbes et al., 2017; Gu et al., 2019). In a study, the sensitivity and specificity of mNGS in invasive fungal disease were 86.76% and 86.98% respectively. mNGS identifies the pathogens in patients with invasive fungal disease more accurately and rapidly than conventional microbiological tests (Wang et al., 2022). This paper summarizes the clinical data of three cases of pediatric CNS IMD to explore diagnostic and treatment processes, aiming to improve the diagnosis and treatment capabilities for this disease.

## 2 Methods

### 2.1 Participants

Three pediatric patients diagnosed with CNS IMD and treated in the PICU of the First Affiliated Hospital of Zhengzhou University from January 2020 to December 2023 were selected for this study. We collected data on the patients’ basic information, clinical characteristics, laboratory and radiological examination results, treatment, and outcomes.

### 2.2 mNGS protocol

The cerebrospinal fluid (CSF) and brain abscess fluid samples were snap-frozen, and stored at − 20 °C until they were delivered to the sequencing center. Total DNA were extracted from the CSF and brain abscess fluid samples with commercial kit after pretreatment with lysozyme and lyticase, then libraries were constructed and sequenced on Ion Proton platform (Life Technologies, USA). Microbiota DNA was extracted using the TIANamp Micro DNA Kit (DP316, TIANGENBIOTECH). After quality control (QC) confirmation, the DNA was randomly fragmented to make a sequencing library. Eligible libraries were sequenced on the NextSeq 550Dx platform (Illumina, USA) with a paired-end sequencing length of 75 bp. Bioinformatics analyses were performed on samples with >10 million raw data. Human host sequences were mapped to the human reference genome (hg19) by removing low-quality and low-complexity regions and removing human sequences using Burrows-Wheeler comparisons. High-quality data were classified by simultaneous alignment to four microbial genome databases (including viruses, bacteria, fungi, and parasites). Databases were downloaded from NCBI (https://www.ncbi.nlm.nih.gov/genomics/). The number of unique alignment reads was calculated and standardized to get the number of reads stringently mapped to pathogen species (SDSMRN) and the number of reads stringently mapped to pathogen genus (SDSMRNG). Retrieve the species information of genomic targets matching the sequence mentioned, and retrieve the specific sequencing reads of all microorganisms and their ratio to the total number of sequences, i.e., relative abundance. Furthermore, using the same wet lab procedures and bioinformatics analyses as the clinical samples, negative controls (sterile deionized water) and positive controls (synthetically known quantities of fragments) are established for each batch of experiments. Finally, the mNGS test report provides the species, the number of specific sequencing reads, and all detected microorganisms (bacteria, fungi, viruses).

### 2.3 Making the final clinical diagnosis

After the results from the mNGS tests are returned, at least four experts from the fields of neurology, radiology, infectious diseases, and microbiology collaboratively review and interpret these reports. Throughout this process, they consider the patient’s clinical background, accompanying symptoms, and other microbiological and laboratory test results. Based on this comprehensive data, this multidisciplinary team assesses whether the patient has CNS IMD and provides a final clinical diagnosis. Once the diagnosis is confirmed, the team members jointly discuss and decide on the most appropriate treatment plan. This multidisciplinary collaboration ensures the accuracy of the treatment decisions and enhances the personalization and specificity of the treatment plan, aiming to provide the best possible medical outcomes for the patient.

## 3 Case descriptions

**Figure 1:**
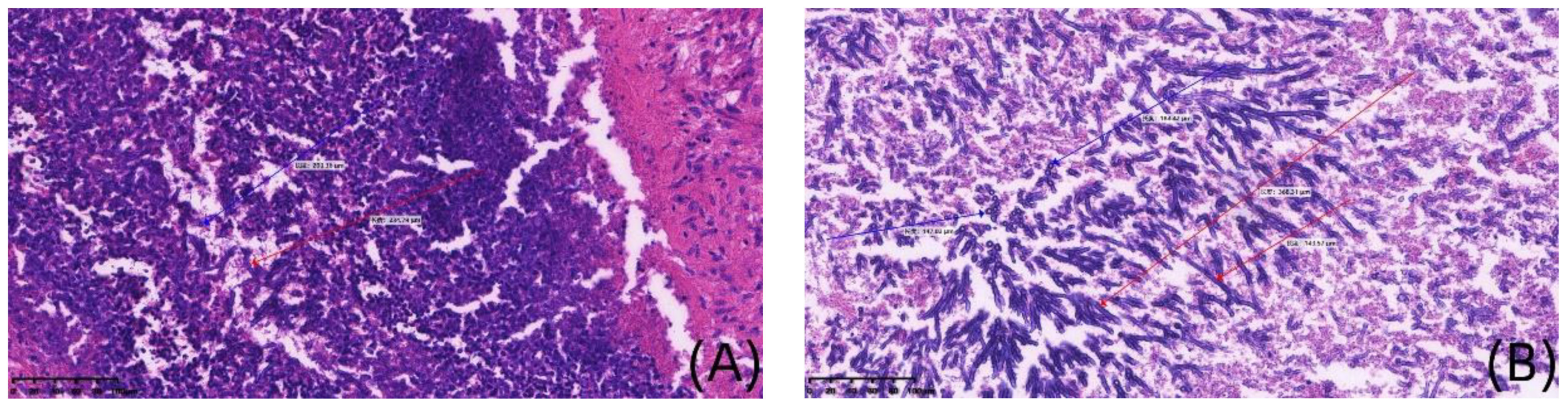
Histopathological staining of brain in case 1(×200). **(A, B)** are detailed histological views at a magnification of 200x, derived from the Image panel, stained with hematoxylin and eosin. Blue arrows show spores, red arrows show mycelium.

**Figure 2:**
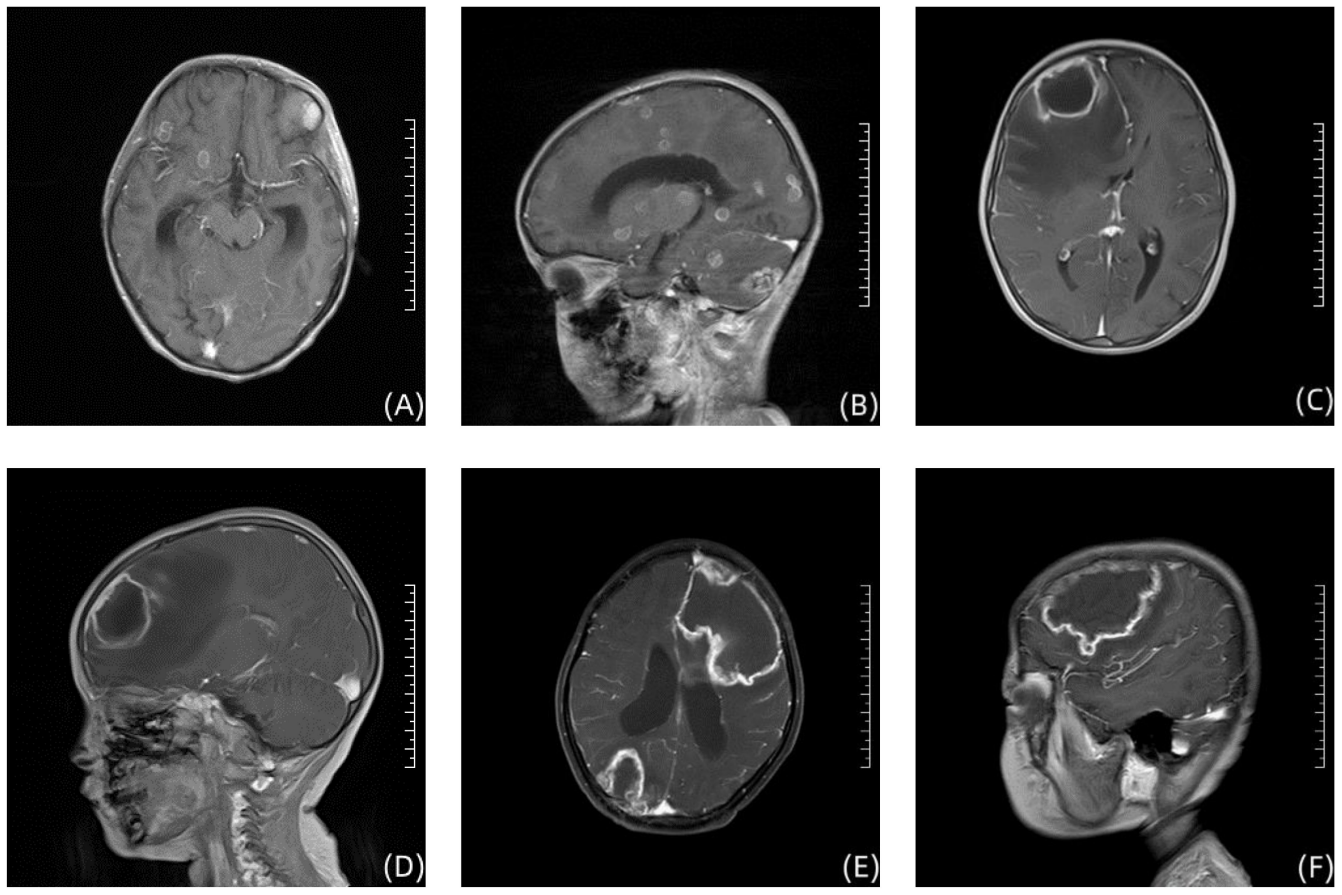
Enhanced T1-weighted brain MRI image in 3 children. **(A, B)** show the Brain imaging of Case 1; **(C, D)** show the Brain imaging of Case 2; **(E, F)** show the Brain imaging of Case 3.

Case 1, a 6-year-old girl, was admitted to the First Affiliated Hospital of Zhengzhou University in November 2020 with “acute liver failure.” There were no significant findings in her personal or family history. After admission, the patient gradually improved following symptomatic treatment including plasma exchange, anti-infection therapy, liver protection, and hemostasis. On the 20th day after admission, the patient began to develop fever, irritability, and seizures. Physical examination revealed muscle strength of 5 in the left limbs, 1 in the right upper limb, and 2 in the right lower limb, with reduced muscle tone in all four limbs. Superficial and tendon reflexes were normal, with a negative Babinski sign and no signs of meningeal irritation. Laboratory tests showed a white blood cell count of 20.30×10^9^/L, neutrophil percentage of 87.2%, lymphocyte percentage of 8.1%, C-reactive protein (CRP) of 30.80mg/L, Procalcitonin (PCT) of 1.770ng/mL, (1, 3)-β-D-glucan(BDG) of 178.40pg/mL and galactomannan (GM) 1.01µg/L. CSF analysis revealed glucose at 4.20mmol/L, chloride at 116mmol/L, lactate dehydrogenase at 39U/L, total protein at 1205mg/L, albumin at 736mg/L, and a white blood cell count of 22.00×10^6^/L; CSF smear did not show abnormal cells; India ink staining did not reveal Cryptococcus. Both CSF culture and CSF immunofluorescence staining were negative. Brain MRI suggested multiple round lesions with long T1 or mixed long T2 signals throughout the brain, surrounded by large patchy long T1 and long T2 signals of edema Brain MRI enhancement suggested multiple abnormal enhancement lesions throughout the brain, suggesting an infectious lesion (**Figures 1A, B**). CSF mNGS did not detect bacteria, fungi, viruses, or special pathogens. Subsequently, the child underwent multiple brain abscess drainage surgeries. Cultures from the intracranial abscess fluid showed the growth of filamentous fungi, indicating *Aspergillus* infection. mNGS results indicated 54 sequences of *Aspergillus fumigatus* (**Table 1**), and the pathology of the excised left frontal inflammatory lesion showed granulomatous inflammation with necrosis, containing fungal hyphae and spores (**Figures 2A, B**). After 20 days of treatment with “voriconazole combined with caspofungin,” the patient’s mental state gradually improved, and her body temperature returned to normal. A six-month follow-up showed no neurological sequelae in the child. Brain MRI of both cerebral hemispheres, the right cerebellar hemisphere, and the vermis showed multiple lesions, with some lesions reduced in size compared to before.

**Table 1:** Summary of metagenomic next-generation sequencing results.

No.	Sample	mNGS results	Sequencing reads	Relative abundance
Case 1	Brain abscess fluid	*Aspergillus fumigatus*	54	41.43%
Case 2	Brain abscess fluid	*Aspergillus fumigatus*	150	99%
Case 3	CSF	*Rhizomucor miehei* *Rhizomucor pusillus*	4 6	NA NA

NA, not applied.

Case 2, a 3-year-old girl, was admitted to the First Affiliated Hospital of Zhengzhou University in September 2022 with “acute B-lymphoblastic leukemia.” There were no significant findings in her personal or family history. She received induction remission therapy with prednisone, vincristine, daunorubicin, and L-asparaginase, supplemented with hydration, antiemetic, cardioprotective, and nutritional support treatments. On the 30th day after starting induction chemotherapy, the patient developed intermittent fever with a peak temperature of 38.1°C and seizures. The physical examination revealed no significant abnormalities in limb muscle strength and muscle tone. Superficial and tendon reflexes were normal, but the Babinski sign was positive bilaterally. The meningeal irritation sign was negative. The treatment approach included vancomycin for infection control, measures to reduce intracranial pressure, fluid resuscitation, and other symptomatic therapies. Laboratory tests showed a white blood cell count of 8.40×10^9^/L, hemoglobin 77g/L, platelets 342×10^9^/L, neutrophil percentage of 89.7%, CRP 43.32mg/L and PCT 0.196ng/mL. CSF analysis revealed a white blood cell count of 1.00×10^6^/L, glucose 4.51mmol/L, chloride 111.8mmol/L, lactate dehydrogenase 9U/L, total protein 146mg/L, and albumin 78.2mg/L; BDG <10pg/mL and GM 0.26µg/L. Brain MRI indicated a mass-like lesion with long T1 mixed long T2 signals in the right frontal lobe and a patchy area with slightly short T2 signals on the left occipital lobe, with a ring-shaped long T2 signal at the edge and a ring-shaped slightly high signal on DWI sequence (**Figures 2C, D**). After a neurosurgical consultation, a cranial abscess was considered, and treatment with vancomycin combined with meropenem for infection was initiated. One week after anti-infective treatment, a follow-up brain MRI scan + enhancement showed that the abscess had slightly increased in size compared to before treatment, with increased surrounding edema. After abscess puncture, mNGS of the abscess fluid indicated *Aspergillus fumigatus*, with a sequence count of 150 (**Table 1**). Vancomycin and meropenem were discontinued, and after 2 weeks of antifungal treatment with voriconazole, the patient no longer had fever. Subsequently, a “right-sided brain abscess drainage surgery” was performed, followed by continued anti-infection treatment, prevention of epileptic seizures, and steroids to reduce cerebral edema. The brain tissue showed chronic suppurative inflammation with granulation tissue proliferation and granuloma formation. Molecular pathology results were negative. At a six-month follow-up, the child had no neurological sequelae. Brain MRI of the right frontal lobe showed abnormal signals, considered postoperative changes and softening lesion formation.

Case 3, a 7-year-old girl, was admitted to the First Affiliated Hospital of Zhengzhou University in December 2021 with “fever for 3 days, lethargy, and weakness in the right limbs for half a day.” There were no significant findings in her personal or family history. In November 2020, she was diagnosed with “acute B-lymphoblastic leukemia” at a local hospital and treated with chemotherapy including doxorubicin, vincristine, L-asparaginase, and dexamethasone. Upon admission, the physical examination revealed: muscle strength of 5 in the left limbs and 4 in the right limbs. Superficial reflexes were normal, but both the knee and Achilles reflexes were hyperactive on both sides. The Babinski sign was positive bilaterally, and the meningeal irritation sign was negative. She was immediately given anti-infection treatment with meropenem combined with vancomycin. Further examinations revealed a white blood cell count of 10.97×10^9^/L, hemoglobin 94.0g/L, platelets 43×10^9^/L; CRP 391.10mg/L, PCT 0.513ng/mL, BDG<10pg/mL and GM 0.45µg/L cerebrospinal fluid (CSF) white blood cell count of 4×10^6^/L, glucose 2.76mmol/L, chloride 118.56mmol/L, lactate 2.1mmol/L, total protein 269mg/L, albumin 168.9mg/L, with no Cryptococcus seen on India ink staining of the CSF. Cranial MRI showed large patchy areas of mixed long T1 and long T2 signals in the left basal ganglia area, left frontal temporal lobe, around the left lateral ventricle, right parieto-occipital lobe, right cerebellar hemisphere, and pontine, and left cerebral peduncle, with mixed diffusive restriction slightly high signals on DWI (**Figures 2E, F**). Chest CT showed patchy high-density shadows in the lower lobe of the left lung, considering inflammation; slight inflammation in the left lung; a small amount of effusion in the left thoracic cavity, left pleural thickening; changes in both kidneys. After 1 week of anti-infection treatment with vancomycin combined with meropenem, the patient still had fever, with a peak temperature of 39.7°C. CSF mNGS indicated the presence of 4 sequences of *Rhizomucor miehei* and 6 sequences of *Rhizomucor pusillus*. Meropenem and vancomycin were discontinued, and she was switched to “amphotericin B combined with posaconazole” for anti-infection treatment. One week later, the patient’s temperature normalized, consciousness cleared, spirit was slightly poor, and vomiting improved. At a six-month follow-up, the child had no neurological sequelae.

## 4 Discussion

*Aspergillus* and *Mucormycetes* spp remain the primary causes of IMD (Pana et al., 2017; Egger et al., 2023). Moulds are ubiquitous organisms that can be found in soil, water, and decaying vegetation (McCarthy et al., 2014). A systematic review of 22 studies found that underlying diseases (such as acute myeloid leukemia, acute lymphoblastic leukemia), allogeneic hematopoietic stem cell transplantation (especially acute and chronic graft-versus-host disease), prolonged neutropenia (>10 days), high-dose steroids, and increased age are the most relevant risk factors for Invasive Fungal Disease in children (Fisher et al., 2018). Outside the clinical setting of cancer patients, diabetes, low birth weight, malnutrition, and liver diseases are risk factors for mold infections (Prakash and Chakrabarti, 2019). The usual portal of entry for these pathogens is the respiratory tract, followed by hematogenous spread to the central nervous system. Alternatively, the central nervous system or paraspinal tissues can be directly infected through surgery or trauma, or possibly spread directly to the central nervous system from the sinuses or mastoid (Miceli, 2019).

In patients with CNS IMD, the initial symptoms are typically nonspecific and may include general weakness, chronic fever, headache, vomiting, subacute dementia, seizures, or neurological deficits (Burgos et al., 2008). Examination of the CSF often reveals mononucleosis (20–500/mL), elevated protein levels, and glucose levels that may be decreased or normal. Imaging findings for CNS IMD present as low signals on T1, high signals on T2, clear contrast enhancement, and a high diffusion coefficient (Jain et al., 2007; Porto et al., 2020). Pathological manifestations range from meningitis and meningoencephalitis to microabscesses, focal necrosis, granulomatous inflammation, large abscesses, vascular infiltration, and infarction. The fundamental lesions are a combination of suppurative and granulomatous inflammation (Singhi and Saini, 2019).

In this study, all three pediatric patients exhibited varying degrees of fever, two experienced seizures, and one presented with limb weakness. Additionally, the routine and biochemical analyses of CSF in these three patients did not match the typical manifestations of CNS IMD. Therefore, any immunocompromised child presenting with subacute to chronic febrile encephalopathy or meningitis, with or without increased intracranial pressure, seizures, orbital pain, and/or serous nasal discharge, should be suspected of having CNS IMD (Groll et al., 2021).

Brain MRI scans revealed varying degrees of abnormal signals in three children, who were ultimately diagnosed with brain abscesses after consultation. While diagnosing brain abscesses is relatively straightforward, identifying the specific causative pathogen is more challenging. Despite initial empirical antimicrobial treatment with meropenem and vancomycin, the condition of the three children remained fluctuating. It was only after the causative microorganisms were identified through mNGS of the abscess fluid or CSF, and antifungal treatment was initiated, that their conditions were effectively managed. This situation underscores the importance of promptly identifying the exact pathogens in patients with compromised immune systems who also present with central nervous system infections, in addition to employing empirical antimicrobial therapy. Such an approach not only enhances the specificity of the treatment but also effectively prevents the recurrence of the condition, accelerating the recovery process for the patients.

In this research, two pediatric patients underwent brain tissue histopathological biopsies, yet only one exhibited a positive result. Although brain tissue histopathology is considered the gold standard, false-negative results still occur, with a diagnostic accuracy of about 78% (Lamoth and Calandra, 2017; Lehrnbecher et al., 2017; Singhi and Saini, 2019). Caudron et al.’s decade-long study showed that invasive mold infections (IMIs) are difficult to diagnose, with a 27% missed diagnosis rate at biopsy and a 53% missed diagnosis rate at autopsy (Caudron et al., 2021).

Among the three children, only Case 1 tested positive for BDG. BDG is a major component of the cell walls of most fungal species, except for those in the *Mucormycetes* and *Cryptococcus* spp. Therefore, a negative BDG result cannot exclude mucormycosis. The sensitivity and specificity of the BDG test in patients with invasive fungal diseases (IFD) range between 75%-83% and 63%-87%, respectively (Calitri et al., 2017; Ferreras-Antolin et al., 2022). This indicates that the BDG test has limited value as an adjunct diagnostic tool for patients with IFD, particularly for those with IMD.

In Case 1, the CSF mNGS testing resulted in a negative outcome. Similarly, in the study by Jin et al., two cases of systemic lupus erythematosus with concurrent Aspergillus brain abscesses also showed negative CSF mNGS results (Jin et al., 2022). Currently, a systematic review on cerebral aspergillosis indicates that 85.1% of patients are diagnosed based on histopathology, while the positivity rate for CSF culture is only 19.1%. This suggests that a negative CSF mNGS result cannot exclude the possibility of an Aspergillus brain abscess, and it is recommended to perform mNGS on abscess fluid after abscess puncture to identify the pathogen. The detection efficacy of mNGS in central nervous system fungal infections can be influenced by multiple factors (Zhang et al., 2020). In the study by Zhang et al., all 13 patients with invasive mucormycosis tested positive using mNGS, suggesting mNGS as a supplementary method for early diagnosis in patients who are unsuitable for histopathological examination or unable to obtain culture specimens (Zhang et al., 2022).

In this study, the results of the G and GM tests for cases 2 and 3 were negative. Although the G and GM test results for case 1 were positive, the initial mNGS result was negative. This, along with the rarity of CNS IMD and the lack of experience among clinicians in this area, led to delayed diagnosis in the three pediatric cases. The inability to diagnose promptly might have impacted the treatment for these patients. Additionally, in this study, mNGS demonstrated its ability to quickly and accurately identify rare and hard-to-culture pathogens, but it also showed some limitations. For instance, mNGS results can be influenced by the type of sample, and empirical treatment might reduce relative abundance and sequencing reads. These factors can decrease diagnostic accuracy and timeliness, further affecting the planning and execution of treatment strategies.

In terms of treatment, the management of fungal CNS infections includes specific antifungal therapy along with supportive measures for related issues such as increased intracranial pressure, metabolic disturbances, management of potential predisposing conditions, and surgical intervention for localized disease, abscesses, or the presence of foreign bodies (such as intracranial shunts) (Spellberg et al., 2009; McCarthy et al., 2014; Chitasombat and Kontoyiannis, 2016; Luckowitsch et al., 2021). All three pediatric patients in this study received aggressive antifungal therapy after the identification of the pathogens and timely surgical interventions, resulting in good therapeutic outcomes with no residual neurological sequelae.

## 5 Conclusion

CNS IMD has an insidious onset and non-specific clinical manifestations, making it challenging to identify the causative pathogen early. The type of sample collected can impact the results of mNGS, complicating the early and definitive identification of the pathogen. To provide a basis for a clear diagnosis, conducting mNGS on appropriately selected samples early can assist in identifying the pathogen, offering a foundation for definitive diagnosis. When necessary, combining this approach with surgical intervention can help improve outcomes by facilitating accurate targeting of the infection and potentially mitigating the progression of the disease.

## Data availability statement

The data supporting the findings of this study are available in the EMBL repository at PRJEB73551. 

## Ethics statement

The studies involving humans were approved by the Ethics Committee of the First Affiliated Hospital of Zhengzhou University. The studies were conducted in accordance with the local legislation and institutional requirements. Written informed consent for participation in this study was provided by the participants’ legal guardians/next of kin. Written informed consent was obtained from the individual(s), and minor(s)’ legal guardian/next of kin, for the publication of any potentially identifiable images or data included in this article.

## Author contributions

EW: Writing – original draft, Writing – review & editing. JN: Writing – original draft. MZ: Data curation, Writing – review & editing. YZ: Data curation, Writing – review & editing. KY: Writing – review & editing. XF: Writing – review & editing. WM: Data curation, Writing – review & editing. LX: Writing – review & editing. PJ: Writing – review & editing. HW: Writing – review & editing.
